# Histological Evaluation of Radiotherapy-Induced Changes in Periodontal Tissues in a Rat Model of Experimental Periodontitis

**DOI:** 10.3390/biology15100803

**Published:** 2026-05-19

**Authors:** Batuhan Hazar Ayşeşek, Buse Başak Feyizoğlu, Vakur Olgaç, İlknur Bingül, Nazlı Ayşeşek, Ülkü Başer, Ayşen Gülden Işık

**Affiliations:** 1 Periodontology Department, Faculty of Dentistry, İstanbul University, 34452 İstanbul, Türkiye; 2 Oral Patology Department, Faculty of Dentistry, Basic Medical Sciences, İstanbul University, 34452 İstanbul, Türkiye; 3 Medical Biochemistry Department, Basic Medical Sciences, Medical Faculty, İstanbul University, 34093 İstanbul, Türkiye; 4 Oral Implantology Department, Faculty of Dentistry, İstanbul University, 34452 İstanbul, Türkiye

**Keywords:** radiotherapy, periodontitis, periodontal ligament, RANKL, osteoprotegerin, rat model, immunohistochemistry, bone remodeling

## Abstract

Background/Objectives: Radiotherapy is a cornerstone of head and neck cancer treatment, but it can adversely affect periodontal tissues by impairing vascularity, cellularity, and healing capacity. The present study aimed to histologically and immunohistochemically evaluate radiotherapy-induced changes in periodontal tissues in a rat model of experimental periodontitis, with particular focus on periodontal ligament width (PerioW) and the RANK/RANKL/OPG axis at different healing stages. Methods: Seventy-two male Sprague–Dawley rats were allocated to three groups: irradiation only (Rt), ligature-induced periodontitis only (Pt), and ligature-induced periodontitis followed by irradiation (PtRt). Experimental periodontitis was induced by placing ligatures around the maxillary first molars for two weeks. On the day of ligature removal, the irradiated groups received a single 20 Gy dose to the head and neck region. Animals were euthanized on days 1, 15, and 30 after ligature removal (n = 8/group/time point). Histomorphometric analysis of PerioW was performed on H&E-stained sections, and immunohistochemical staining was used to quantify RANK, RANKL, and OPG expression. Results: On day 1, PerioW did not differ significantly among groups, although the PtRt group had the highest mean. A significant intergroup difference was observed for RANKL, with higher expression in PtRt than in Pt. On day 15, PerioW differed significantly among groups, with the lowest value in Rt and the highest in PtRt; the Rt–PtRt comparison was significant. At this time point, RANK, RANKL, OPG, and the RANKL/OPG ratio showed no significant intergroup differences. On day 30, no significant intergroup differences were found for PerioW or immunohistochemical parameters; however, PtRt continued to show the highest PerioW and OPG values and the lowest RANKL/OPG ratio. Conclusions: Radiotherapy superimposed on periodontitis enhanced early pro-resorptive signaling and delayed structural normalization of periodontal tissues. Although late increases in OPG suggested a compensatory response, this appeared insufficient to fully reverse radiation-associated periodontal alterations. These findings support the importance of controlling periodontal inflammation before radiotherapy to reduce subsequent periodontal tissue damage.

## Simple Summary

Radiotherapy is widely used to treat cancers in the head and neck, but it can also harm healthy tissues in the mouth. One of the most vulnerable areas is the tissue that supports the teeth, which may lead to long-term oral problems. Gum disease is another common condition that weakens these tissues through inflammation. In this study, we explored what happens when these two conditions occur together. Using an experimental model, we examined how radiation affects bone and regeneration around teeth in the presence of periodontal disease. We observed that radiation increased tissue damage in the early stages and slowed healing over time. Although the body tried to protect the bone in later stages, this response was not enough to fully restore normal conditions. These findings suggest that patients receiving radiotherapy may face greater risks to their oral health, especially if gum disease is already present. A better understanding of these effects may help improve prevention and treatment approaches for these patients.

## 1. Introduction

Periodontitis is the inflammation of the periodontal tissues due to multifactorial causes in the oral environment. Periodontal disease is initiated by a bacterial biofilm that elicits a host inflammatory response within the periodontal tissues, ultimately resulting in periodontal ligament detachment from the cementum, alveolar bone resorption, and gingival recession [1]. The progression of periodontal disease is strongly influenced by the complex interaction between periopathogenic microorganisms and the host immune system. Although the immune response is primarily protective, an exaggerated and dysregulated inflammatory reaction contributes substantially to connective tissue destruction and alveolar bone loss [2]. 

Early-stage inflammation is driven by immune signals like Leukotriene B4 and Tumor Necrosis Factor Alpha (TNF-α), while cytokines such as IL-1 and IL-6 fluctuate in the tissue and saliva, providing potential biomarkers for predicting periodontitis [3,4]. Proinflammatory cytokines, along with the activation of NF-kB and RANKL-mediated osteoclastogenesis, further contribute to bone destruction, while OPG serves as a protective factor, safeguarding the cementum from root resorption [3,5]. Dysregulation of these inflammatory and immune pathways leads to chronic inflammation, tissue destruction, and disease [6,7,8].

One of the possibilities that can alter the immune response is radiation. Radiation therapy, especially in head and neck cancer treatment, can severely affect oral tissues, leading to complications such as mucositis, fibrosis, fungal infections, pain, and taste disturbances. In cases of higher radiation doses, salivary gland hypofunction and xerostomia (dry mouth) can occur, significantly impacting oral functions, general health, and overall quality of life [9,10]. The periodontium is highly sensitive to radiation. Post-radiotherapy, the risk of periodontal attachment loss increases due to reduced vascularity and cellular structure in the periodontal membrane. This process is accompanied by changes in biomarker levels in both saliva and the bloodstream [11]. As many studies suggest, eliminating periodontal infections prior to radiation therapy is crucial to reducing these risks [12].

In our study, we aimed to analyze the early and late effects of radiation on periodontitis-induced rats. We sought to observe changes in periodontal tissues in rats with induced periodontitis that were also subjected to radiation. Our hypothesis was that radiation would lead to distinct changes in specific biomarkers in tissues at different stages of the post-radiation period.

## 2. Materials and Methods

### 2.1. Study Design and Experimental Groups

This in vivo study was conducted using a ligature-induced periodontitis model with/without irradiation. A total of 72 male Sprague–Dawley rats between 10 and 12 weeks old (200–250 g) were allocated to three experimental groups and followed across three euthanasia time points (days 1, 15, and 30) after ligature removal. At each time point, eight rats per group were euthanised.

**Figure 1:**
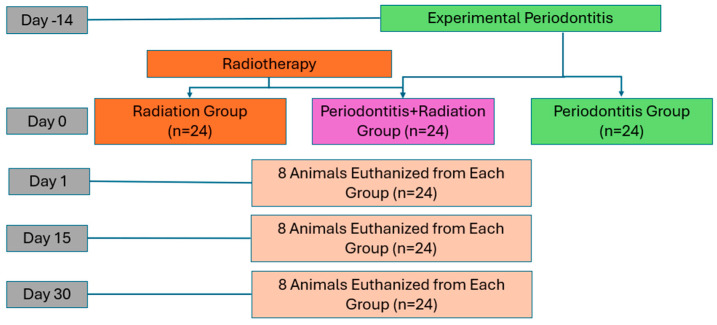
Schematic overview of the experimental design, group allocation, and euthanasia time points (days 1, 15, and 30). (n = 8/group/time point; total n = 72).

The groups were defined as follows (Figure 1):

1. Control-Irradiated (Rt): irradiation without periodontitis induction.
2. Periodontitis (Pt): ligature-induced periodontitis without irradiation.
3. Periodontitis + Irradiation (PtRt): ligature-induced periodontitis followed by irradiation.
4. Ligatures were placed for two weeks to induce experimental periodontitis [13]. After clinical signs of periodontitis were observed, ligatures were removed, and animals were randomized into irradiated and non-irradiated periodontitis groups.

### 2.2. Ethical Approval and Animal Housing

All procedures were performed in accordance with institutional and international guidelines for animal research and were approved by the Animal Experimentation Ethics Committee of İstanbul University (Approval No: 303112). All experiments were conducted in line with international ethical principles for the care and use of laboratory animals, and the manuscript was prepared in conformity with the ARRIVE guidelines [14]. Animals were housed under standard laboratory conditions and fed a normal diet with ad libitum access to water throughout the study. During the 14-day ligature period, animals were monitored daily to ensure correct ligature placement and successful induction of periodontitis. After the experimental procedures, animals were checked at regular intervals until the designated euthanasia time points.

### 2.3. Sample Size Calculation

A power analysis indicated that a sample size of n = 8 per group would provide 80% power to detect differences with a significance level of α = 0.05 and an effect size (d) of 0.68. As the data followed a normal distribution and had a coefficient of variation below 20%, group differences were analyzed using ANOVA and the Tukey HSD multiple comparison test in SPSS 17.0 (SPSS Inc.; IBM Company, Chicago, IL, USA). Data were expressed as mean values with minimum and maximum values, and ±SD. A *p*-value of <0.05 was considered statistically significant. Considering three experimental groups and three euthanasia time points (1st, 15th, 30th days) with eight animals per group at each time point, a total of 72 rats were included. Animals were randomly assigned to experimental groups using a simple randomization approach. Group allocation was performed prior to experimental procedures.

### 2.4. Surgical Procedures

**Figure 2:**
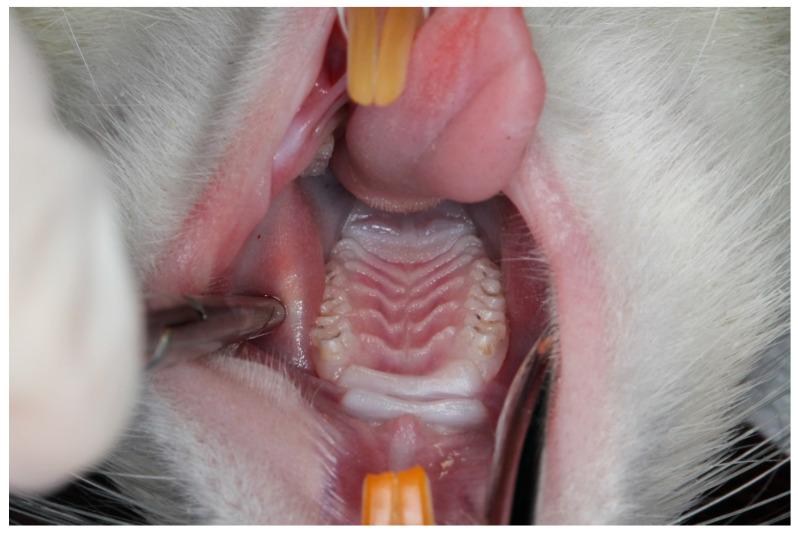
Representative photograph of the rat maxilla showing the anatomical region used for ligature placement around the cervical area of the maxillary first molar to induce experimental periodontitis.

**Figure 3:**
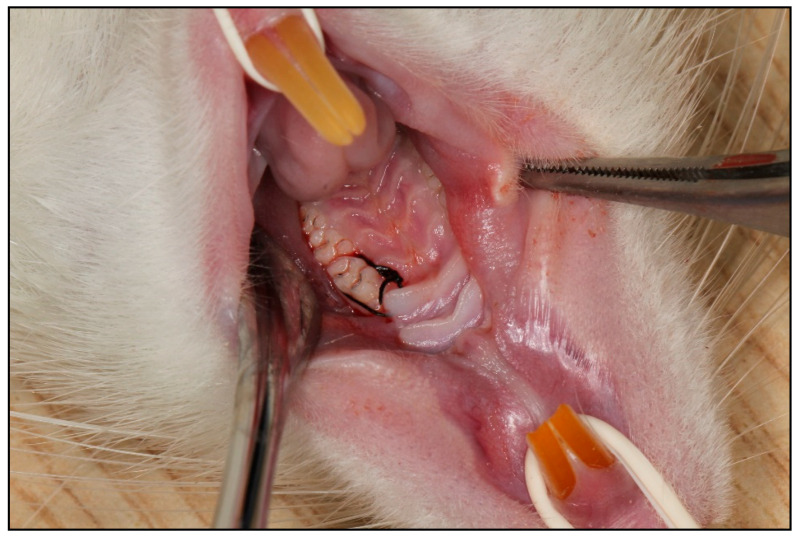
Intraoral representative image demonstrating the ligature placement technique around the maxillary first molar. The 5/0 sterilized silk suture was configured in an “O” shape and secured with a knot on the mesial surface of the tooth.

All surgical interventions were performed under general anesthesia induced by intraperitoneal administration of 50 mg/kg ketamine HCl (Ketalar^®^ Flakon, Pfizer, Istanbul, Turkey) combined with 10 mg/kg xylazine (Rompun^®^ Flakon, Bayer Türk Kimya Sanayi Ltd. Şti., Istanbul, Turkey). Experimental periodontitis was induced using a ligature-based model; under anesthesia, a 5/0 sterilized silk suture (Doğsan, Istanbul, Türkiye) was placed around the cervical region of the maxillary first molar, configured in an “O” shape, and secured with a knot positioned on the mesial surface of the tooth. To facilitate accurate placement, a slight separation of the interproximal space between the first and second molars was created using a periodontal probe (Hu-Friedy, Chicago, IL, USA). Ligatures were maintained for two weeks in accordance with the protocol described by de Molon et al. [15], and were inspected every other day and repositioned when necessary to ensure stable placement throughout the induction period (Figure 2 and Figure 3).

Postoperative analgesia and antimicrobial prophylaxis were provided to reduce animal discomfort and minimize infection risk. Accordingly, tramadol HCl (Tramadolor 100 mg ampoule, Sandoz İlaç San. ve Tic. A.Ş., Istanbul, Turkey) and gentamicin (Gentamed 20 mg ampoule, Koçak Farma İlaç ve Kimya San. A.Ş., Istanbul, Turkey) were administered intraperitoneally twice daily for 3 days during the postoperative period.

### 2.5. Radiotherapy

The irradiated groups (Rt and PtRt) received a single fraction of radiation on the day of ligature removal (40–60 min after removal). For irradiation, rats were anesthetized and immobilized to minimize motion during dose delivery and to ensure reproducible positioning. Animals were positioned dorsoventrally, and irradiation was confined to the head and neck region. Each animal was irradiated individually. Radiation was delivered using a medical linear accelerator (Varian Clinac–DBX, Palo Alto, CA, USA) at a 0° gantry angle with 6 MV X-rays and a focus–axis distance (FAD) of 100 cm. The irradiation field size was 10 cm × 20 cm, and the source-to-skin distance was 98.5 cm. A total dose of 20 Gy was administered as single-dose head-and-neck irradiation, consistent with previously described protocols [16].

### 2.6. Euthanasia

At the predetermined time points (days 1, 15, and 30 following ligature removal), animals were deeply anesthetized via intraperitoneal administration of 50 mg/kg ketamine HCl (Ketalar^®^ Flakon, Pfizer, Turkey) and 10 mg/kg xylazine (Rompun^®^ Flakon, Bayer Türk Kimya Sanayi Ltd. Şti., Turkey). Following confirmation of adequate anesthesia, the animals were euthanized, and the maxillae were carefully dissected. Harvested specimens were immediately fixed in 10% buffered formalin (Birpa Formaldehit, Birpa Kimyevi Maddeler Paz. ve Tic. Ltd. Şti., Yenimahalle, Ankara, Turkey) for subsequent histological processing.

### 2.7. Histomorphometric and Immunohistochemical Analysis

At each predetermined euthanasia time point (days 1, 15, and 30 following ligature removal), the maxillae were carefully excised and immediately fixed in 10% neutral-buffered formalin for 1 week. Specimens were subsequently decalcified in a solution containing 50% formic acid and 20% sodium citrate until adequate decalcification was achieved. Following decalcification, the ligature-applied (experimental periodontitis) side of each maxilla was processed for histological evaluation and sagittally sectioned through the maxillary first molar region. Tissues were dehydrated through graded alcohols, cleared, and embedded in paraffin. Paraffin blocks were evaluated for the cellular expression of RANK, OPG, and RANKL following the protocol provided by the manufacturer (Thermo Fisher Scientific Inc., Waltham, MA, USA). RANK antibody [1:10–500] (catalog no. MA5-16,153), RANKL antibody [1:200] (catalog no. MA5-16,156), and OPG antibody [1:10–500] (catalog no. MA5-15,960) (all from Thermo Fisher Scientific Inc., Waltham, MA, USA; all diluted as recommended by product information sheets) were used. The sections were pretreated with a blocking solution to avoid nonspecific reactions. Serial sections (3–5 μm) were prepared and stained with hematoxylin and eosin (H&E) for light microscopic assessment.

**Figure 4:**
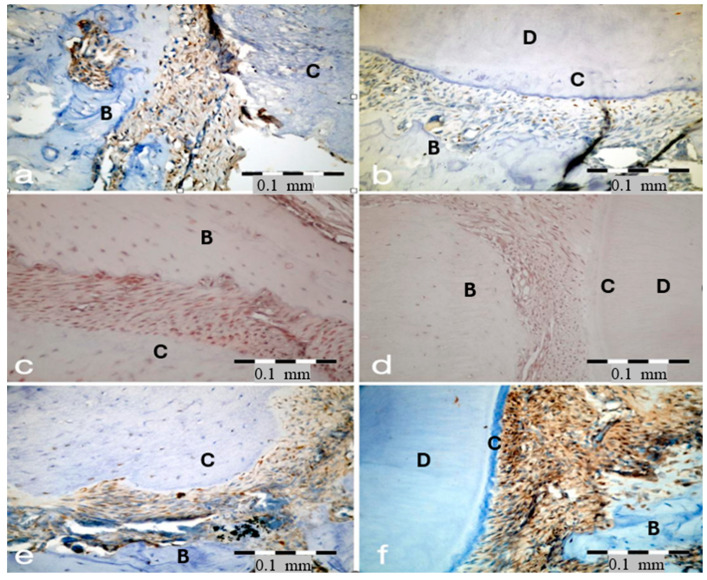
Representative immunohistochemical staining of RANKL, RANK, and OPG in periodontal tissues. Panels (**a**,**b**) demonstrate RANKL expression, (**c**,**d**) RANK expression, and (**e**,**f**) OPG expression. For better visualization, key anatomical structures were indicated in black: bone (B), cementum (C), and dentin (D).

Digital images were acquired and analyzed at ×400 magnification using Olympus Viewer 3 imaging software. For histomorphometric evaluation, three standardized regions of interest (ROIs) were selected at defined anatomical locations along the periodontal ligament of the maxillary first molar. These regions corresponded to the coronal, middle, and apical thirds of the periodontal ligament space. PerioW was measured within these regions using digital image analysis software under 400× magnification. Due to variations in periodontal ligament width within the same region, measurements were performed at the widest point of the periodontal ligament space perpendicular to the root surface. A standardized perpendicular line was drawn from the root surface to the adjacent alveolar bone, and the distance between these two structures was recorded as the PerioW. For each region of interest, three measurements were taken, and the mean value was used for statistical analysis. Immunohistochemical evaluation was performed using a semi-quantitative analysis method. Positively stained cells were identified based on the presence of distinct brown cytoplasmic staining. For each section, three standardized regions of interest were selected, and the number of positively stained cells was counted under 400× magnification using a light microscope. Only cells showing clear and specific staining were included in the analysis, while background or nonspecific staining was excluded. The mean value of the three regions was used for statistical analysis (Figure 4).

For each marker, representative images of high and low immunoreactivity are presented to illustrate differences in staining intensity. Strong positive staining was predominantly observed in inflammatory connective tissue areas, whereas weaker staining was detected in regions with lower cellular activity. Positive immunoreactivity is indicated by brown DAB staining. All sections were counterstained with hematoxylin and examined at ×400 magnification. Scale bar = 0.1 mm.

### 2.8. Statistical Analysis

Data normality was assessed using the Shapiro–Wilk test. As the data were normally distributed and demonstrated a coefficient of variation below 20%, group comparisons were performed using analysis of variance (ANOVA) followed by Tukey’s HSD post hoc test for multiple comparisons. Statistical analyses were conducted using SPSS v17.0 (SPSS Inc.; IBM Company, Chicago, IL, USA). Data were presented as mean and standard deviation, together with minimum and maximum values. A *p*-value < 0.05 was considered statistically significant.

## 3. Results

### Histomorphometric Analysis

**Figure 5:**
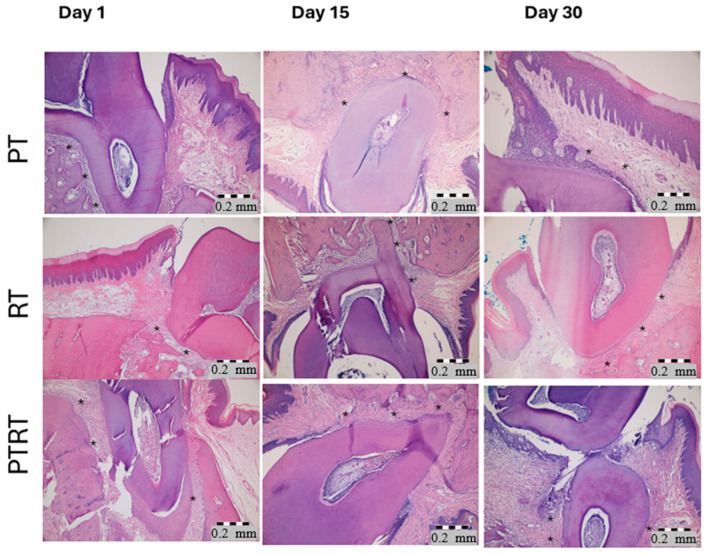
Representative histological images of periodontal tissues from the Pt, Rt, and PtRt groups at Days 1, 15, and 30. Asterisks (*) indicate the specific locations where periodontal ligament width (PerioW) measurements were performed at 400× magnification. All sections were stained with hematoxylin and eosin (H&E) and examined under light microscopy. Scale bar = 0.2 mm.

Histologically, there were no significant differences in PerioW values on day 1. However, the PtRt group showed the highest mean PerioW values (11.40 ± 1.72) compared with the Pt group (9.87 ± 0.99) and the Rt group (10.21 ± 1.44). Immunohistochemically, a significant intergroup difference was detected for RANKL (ANOVA; *p* = 0.03) on day 1; post hoc analysis showed higher RANKL in the PtRt group (26.12 ± 5.64) than the Pt group (17.50 ± 5.52) (Tukey *p* = 0.032). In contrast, RANK (Pt: 23.50 ± 6.04; Rt: 27.87 ± 6.33; PtRt: 28.62 ± 6.88), OPG (Pt: 18.25 ± 4.13; Rt: 20.25 ± 2.86; PtRt: 21.25 ± 2.49), and the RANKL/OPG ratio (Pt: 0.98 ± 0.37; Rt: 1.20 ± 0.23; PtRt: 1.25 ± 0.32) did not show statistically significant differences among groups on Day 1 (ANOVA; *p* > 0.05) (Table 1) (Figure 5).

On Day 15, PerioW differed significantly among groups (ANOVA, *p* = 0.011). The Rt group exhibited the lowest mean PerioW value (9.66 ± 1.25). In contrast, the PtRt group showed a higher value (11.33 ± 1.09), and post hoc analysis confirmed a significant difference between Rt and PtRt (Tukey, *p* = 0.014). Immunohistochemically, RANK (Pt: 37.87 ± 3.22; Rt: 33.25 ± 13.13; PtRt: 41.12 ± 12.02), RANKL (Pt: 34.12 ± 4.64; Rt: 32.50 ± 9.39; PtRt: 37.50 ± 8.81), OPG (Pt: 34.75 ± 3.57; Rt: 29.25 ± 4.52; PtRt: 30.12 ± 7.79) and the RANKL/OPG ratio (Pt: 0.98 ± 0.09; Rt: 1.11 ± 0.25; PtRt: 1.27 ± 0.22) did not show statistically significant differences among groups on Day 15 (ANOVA; *p* > 0.05) (Table 2).

On Day 30, PerioW did not differ significantly among groups (ANOVA, *p* > 0.05); nevertheless, the PtRt group again exhibited the highest mean PerioW value (11.77 ± 2.16) compared with the Pt (9.85 ± 1.05) and Rt groups (10.40 ± 1.29). Immunohistochemically, RANK (Pt: 33.37 ± 4.06; Rt: 36.50 ± 10.30; PtRt: 41.37 ± 12.38), RANKL (Pt: 32.00 ± 5.34; Rt: 30.37 ± 8.08; PtRt: 31.50 ± 8.96), OPG (Pt: 27.12 ± 6.51; Rt: 29.12 ± 8.28; PtRt: 36.12 ± 9.74) and the RANKL/OPG ratio (Pt: 1.20 ± 0.15; Rt: 1.07 ± 0.26; PtRt: 0.89 ± 0.20) did not show statistically significant differences among groups on Day 30 (ANOVA; *p* > 0.05) (Table 3).

## 4. Discussion

Radiotherapy is an essential component of head and neck cancer treatment; however, it may adversely affect oral and periodontal tissues through alterations in vascularity, cellularity, collagen metabolism, and tissue remodeling capacity, thereby creating a hypovascular, hypocellular, and hypoxic microenvironment that is more susceptible to infection, attachment loss, and delayed healing [12,17,18]. In clinical settings, irradiated patients have been reported to show progressive periodontal deterioration, including increased pocket depth, attachment loss, and widening of the periodontal ligament space, particularly when periodontal health is already compromised before treatment [18,19]. Experimental evidence likewise suggests that irradiation can aggravate periodontal destruction in the presence of pre-existing inflammation by increasing oxidative stress and tissue breakdown [20]. In parallel, alveolar bone resorption in periodontitis is strongly regulated by the RANK/RANKL/OPG system, in which increased RANKL activity and insufficient OPG-mediated inhibition promote osteoclast differentiation and bone loss [21,22]. Although both radiotherapy-related tissue injury and periodontitis-associated osteoimmune activation have been described separately, histologic and immunohistochemical data clarifying how irradiation influences alterations in the periodontal ligament and the RANK/RANKL/OPG axis in inflamed periodontal tissues remain limited [20]. Therefore, the aim of the present study was to investigate and compare the histological alterations in periodontal ligament width and the immunohistochemical expression patterns of RANK, RANKL, and OPG at Days 1, 15, and 30 of the healing period in rats with experimental periodontitis following radiotherapy. Unlike previous studies that primarily focused on radiation-induced changes in isolation, the present study evaluates the combined and time-dependent effects of radiotherapy and periodontal inflammation, revealing a dynamic interplay between early osteoclastogenic activation and delayed compensatory responses.

In clinical practice, radiotherapy is typically administered in a fractionated schedule; however, in experimental animal models, single-dose irradiation is frequently preferred. This is mainly due to the higher regenerative capacity and faster healing response observed in rodents, which may reduce the cumulative effects of fractionated doses. In the present study, a single 20 Gy dose was used to ensure a consistent and measurable biological response in periodontal tissues. Although this model allows for controlled evaluation of radiation-induced changes, it does not fully replicate the fractionated regimens used in clinical settings, which should be considered when interpreting the results.

Although PerioW values did not differ significantly among groups on Day 1 (*p* > 0.05), the highest mean value observed in the PtRt group (11.40 ± 1.72), compared with the Pt (9.87 ± 0.99) and Rt groups (10.21 ± 1.44), suggests an early tendency toward periodontal ligament space widening when periodontitis and irradiation coexist. This finding is consistent with the concept that widening of the periodontal ligament space reflects disrupted periodontal homeostasis, altered vascularity, and delayed connective tissue remodeling following irradiation. Chan et al. reported mandibular changes in 60% of patients after radiotherapy, 88% of which corresponded to widening of the periodontal ligament space, and further showed that this alteration developed significantly earlier in segments exposed to ≥45 Gy (*p* < 0.001) [23]. Within this context, although the present study evaluated histologic changes in a short-term rat model rather than late radiographic findings in humans, the increased PerioW value detected in the PtRt group on Day 1 may represent an early microscopic counterpart of these post-radiotherapy periodontal alterations.

Immunohistochemically, the findings further suggest that the earliest measurable effect of combined periodontitis and radiotherapy is not a fully established structural divergence, but rather an increase in pro-resorptive signaling. Representative immunohistochemical images illustrating variations in RANKL, RANK, and OPG expression are shown in Figure 4. Specifically, RANKL expression was significantly higher in the PtRt group (26.12 ± 5.64) than in the Pt group (17.50 ± 5.52) on Day 1 (*p* = 0.032), whereas no significant differences were observed in RANK, OPG, or the RANKL/OPG ratio (*p* > 0.05). This pattern is in agreement with previous experimental evidence. Köse et al. demonstrated that the addition of radiotherapy to experimental periodontitis significantly increased periodontal destruction and anti-RANKL-positive osteoclast density (*p* < 0.05), while Alwood et al. showed that irradiation alone was sufficient to trigger a rapid pro-osteoclastogenic response, resulting in a 4.1-fold increase in RANKL expression by Day 1, whereas OPG did not change significantly [20,24]. Taken together, these findings suggest that, in the early phase, radiotherapy superimposed on periodontitis may enhance RANKL-mediated pro-resorptive activity rather than simultaneously altering the entire RANK/RANKL/OPG axis.

In contrast, RANK, RANKL, OPG, and the RANKL/OPG ratio did not differ significantly among groups on Day 15 (*p* > 0.05). This apparent discrepancy between morphologic significance and immunohistochemical non-significance is biologically plausible and may indicate that the early pro-resorptive signaling observed on Day 1 had evolved into a more heterogeneous remodeling phase by Day 15. In this context, Giannopoulou et al. demonstrated that although RANK and RANKL are broadly expressed in chronic periodontitis, OPG expression is less consistent, suggesting that these markers may not reliably distinguish intermediate stages of periodontal healing or remodeling [21]. Likewise, Wu et al. showed that pro-resorptive activity may predominate without parallel alterations in all regulatory markers [25]. In addition, De Almeida et al. suggested that the 15-day period may represent a transitional stage between active tissue destruction and partial regulatory recovery [26]. Taken together, the present findings indicate that radiotherapy superimposed on periodontitis may delay structural normalization of the periodontal ligament by Day 15, even in the absence of statistically significant intergroup differences in the RANK/RANKL/OPG axis.

By Day 15, PerioW emerged as the most discriminative histologic parameter, with a significant intergroup difference (ANOVA, *p* = 0.011) driven primarily by the lower value in the Rt group (9.66 ± 1.25) and the higher value in the PtRt group (11.33 ± 1.09), while the Pt group remained intermediate. This pattern suggests that, at the mid-healing stage, the combined effect of periodontitis and radiotherapy is manifested more clearly at the level of periodontal structure than through isolated immunohistochemical endpoints. From a biological perspective, widening of the periodontal ligament space at this stage is more likely to reflect persistent tissue disorganization than ongoing acute inflammation alone, including residual edema, compromised vascular support, collagen disarray, and delayed re-establishment of normal periodontal architecture. This interpretation is consistent with experimental radiation models showing that the irradiated periodontal membrane becomes fibrotic, hypocellular, poorly organized, and infiltrated by mononuclear inflammatory cells, while adjacent soft tissues display reduced vascularity and denser collagen networks [16]. It is also concordant with clinical observations that a widened periodontal ligament space is a common post-radiotherapy jaw change rather than a nonspecific inflammatory finding; notably, Chan et al. [23] reported mandibular changes in 75 of 126 irradiated patients (60%), of which 66 of 75 (88%) were widened periodontal ligament space, with a median detection time of 29 months.

In contrast, RANK, RANKL, OPG, and the RANKL/OPG ratio did not differ significantly among groups on Day 15, despite numerically higher RANK (41.12 ± 12.02), RANKL (37.50 ± 8.81), and RANKL/OPG ratio (1.27 ± 0.22) values in the PtRt group. This apparent divergence between morphologic significance and immunohistochemical non-significance is biologically plausible and may indicate that the early pro-resorptive signaling detected on Day 1 had, by Day 15, transitioned into a more heterogeneous remodeling phase in which structural consequences remained evident even though marker expression was no longer sharply separated between groups. This interpretation is supported by Giannopoulou et al., who showed that in chronic periodontitis, more than 60% of the inflammatory infiltrate stained positive for RANK in 64.3% of cases and for RANKL in 57.1% of cases, whereas OPG positivity in more than 60% of inflammatory cells was observed in only 35.7% of cases, indicating that RANK and RANKL remain broadly expressed during active periodontal remodeling while OPG is less consistently upregulated [21].

Although no significant difference was detected in PerioW values on Day 30 (*p* > 0.05), the PtRt group (11.77 ± 2.16) continued to exhibit the highest mean value compared to the Pt group (9.85 ± 1.05) and the Rt group (10.40 ± 1.29). This persistence suggests that structural changes induced by radiotherapy, when superimposed on periodontitis, may resist complete normalization even during the late stages of healing. Immunohistochemically, the downward trend in RANKL expression across all groups relative to Day 15 indicates attenuation of the acute inflammatory response and transition toward a chronic remodeling phase. Morita et al. reported that decreased RANKL expression was associated with reduced local tissue inflammation and a lower risk of osteonecrosis, and our findings appear consistent with this pattern of molecular stabilization [27].

On the other hand, the observation that OPG reached its highest numerical value in the PtRt group on Day 30 (36.12 ± 9.74), resulting in the lowest RANKL/OPG ratio (0.89 ± 0.20) among the groups, suggests that the tissue mounted a strong counter-regulatory response to suppress ongoing destruction in the late phase. Kim et al. similarly reported that OPG levels continued to increase until Day 21 following low-dose gamma radiation [28]. However, despite this apparent improvement in the RANKL/OPG balance, the persistently elevated PerioW value in the PtRt group reinforces the notion that radiotherapy may leave microscopic and biomechanical damage within the periodontal ligament that is either slow to repair or not fully reversible. In this respect, Sroussi et al.emphasized that hypocellular and fibrotic alterations induced by radiotherapy in oral tissues may contribute to the persistence of structural periodontal-like defects even after molecular markers begin to normalize [29].

This study has several limitations that should be considered when interpreting the findings. First, the absence of a healthy, untreated control group limits the ability to establish baseline physiological values, and therefore comparisons were made between experimental conditions rather than normal tissue. Second, only male animals were used to reduce biological variability; however, this may limit the generalizability of the findings, as sex-related differences can influence inflammatory responses, bone remodeling, and radiation sensitivity. In addition, direct functional markers of osteoclast activity, such as TRAP staining, were not included, which may limit the interpretation of bone resorption at a functional level. Although the sample size was determined based on power analysis, variability observed in certain parameters may have affected statistical power, and thus some non-significant findings should be interpreted with caution, as they may reflect biological variability rather than the absence of an effect. Finally, interaction effects between treatment group and time were not formally evaluated, as different animals were used at each time point. Future studies incorporating healthy control groups, both sexes, functional validation methods, and more comprehensive statistical models may provide a more detailed understanding of periodontal tissue responses to radiotherapy.

## 5. Conclusions

This study suggests that radiotherapy may influence periodontal bone metabolism and may be associated with increased osteoclastogenic activity in the presence of periodontitis. While periodontitis alone followed a predictable inflammatory pattern with peak activity at Day 15 and partial late compensation, the combination with radiation was associated with earlier RANK/RANKL activation and a greater tendency toward imbalance in the RANK–RANKL–OPG system. Although OPG increased over time as a compensatory response, this may not have been sufficient to fully counteract the observed structural alterations, particularly in the combined group. Radiation alone was associated with moderate changes; however, when combined with inflammation, it appeared to produce additive or enhanced effects, leading to increased and more extended alterations in bone remodeling. These findings highlight the potential importance of controlling periodontal inflammation prior to radiotherapy to potentially reduce radiation-associated bone changes and long-term periodontal effects.

## Figures and Tables

**Table 1:** Histological and immunohistochemical analysis of periodontal tissues for Day 1.

Groups	PerioW	RANK	RANKL *	OPG	RANKL/OPG
Pt	9.87 ±0.99	23.50 ± 6.04	17.50 ± 5.52 ^a^	18.25 ± 4.13	0.98 ± 0.37
Rt	10.21 ± 1.44	27.87 ± 6.33	24.50 ± 5.92	20.25 ± 2.86	1.20 ± 0.23
PtRt	11.40 ± 1.72	28.62 ± 6.88	26.12 ± 5.64 ^a^	21.25 ± 2.49	1.25 ± 0.32

* ANOVA; *p* = 0.03; ^a^ Tukey *p* = 0.032.

**Table 2:** Histological and immunohistochemical analysis of periodontal tissues for Day 15.

Groups	PerioW *	RANK	RANKL	OPG	RANKL/OPG
Pt	10.25 ± 0.99	37.87 ± 3.22	34.12 ± 4.64	34.75 ± 3.57	0.98 ± 0.09
Rt	9.66 ± 1.25 ^b^	33.25 ± 13.13	32.50 ± 9.39	29.25 ± 4.52	1.11 ± 0.25
PtRt	11.33 ± 1.09 ^b^	41.12 ± 12.02	37.50 ± 8.81	30.12 ± 7.79	1.27 ± 0.22

* ANOVA, *p* = 0.011; ^b^ Tukey, *p* = 0.014.

**Table 3:** Histological and immunohistochemical analysis of periodontal tissues for Day 30.

Groups	PerioW	RANK	RANKL	OPG	RANKL/OPG
Pt	9.85 ± 1.05	33.37 ± 4.06	32.00 ± 5.34	27.12 ± 6.51	1.20 ± 0.15
Rt	10.40 ± 1.29	36.50 ± 10.30	30.37 ± 8.08	29.12 ± 8.28	1.07 ± 0.26
PtRt	11.77 ± 2.16	41.37 ± 12.38	31.50 ± 8.96	36.12 ± 9.74	0.89 ± 0.20

## Data Availability

The data that support the findings of this study are available from the corresponding author upon request.

## References

[1] Löe H., Theilade E., Wright W.H., Jensen S.B. (1966). Experimental gingivitis in man. II. A longitudinal clinical and bacteriological investigation. J. Periodontal Res..

[2] Slots J. (2017). Periodontitis: Facts, fallacies and the future. Periodontol. 2000.

[3] Kim J.-Y., Kim K.-R., Kim H.-N. (2021). The potential impact of salivary IL-1 on the diagnosis of periodontal disease: A pilot study. Healthcare.

[4] Graves D.T., Cochran D. (2003). The contribution of interleukin-1 and tumor necrosis factor to periodontal tissue destruction. J. Periodontol..

[5] Takayanagi H. (2007). Osteoimmunology: Shared mechanisms and crosstalk between the immune and bone systems. Nat. Rev. Immunol..

[6] Liu W., Zhang X. (2015). Receptor activator of nuclear factor-κB ligand (RANKL)/RANK/osteoprotegerin system in bone and other tissues (review). Mol. Med. Rep..

[7] Sakata M., Shiba H., Komatsuzawa H., Fujita T., Ouhara K., Kajiya M., Takeda K., Kurihara H. (1999). Expression of osteoclast differentiation factor (RANKL) in periodontal disease. J. Dent. Res..

[8] Wada T., Nakashima T., Hiroshi N., Penninger J.M. (2001). RANKL–RANK signaling in osteoclastogenesis and bone disease. Trends Mol. Med..

[9] Langendijk J.A., Doornaert P., Verdonck-de Leeuw I.M., Leemans C.R., Aaronson N.K., Slotman B.J. (2008). Impact of late treatment-related toxicity on quality of life among patients with head and neck cancer treated with radiotherapy. J. Clin. Oncol..

[10] Jensen S.B., Pedersen A.M.L., Vissink A., Andersen E., Brown C.G., Davies A.N., Dutilh J., Fulton J.S., Jankovic L., Lopes N.N.F. (2010). A systematic review of salivary gland hypofunction and xerostomia induced by cancer therapies: Prevalence, severity and impact on quality of life. Support. Care Cancer.

[11] Epstein J.B., Lunn R., Le N., Stevenson-Moore P. (1998). Periodontal attachment loss in patients after head and neck radiation therapy. Oral Surg. Oral Med. Oral Pathol. Oral Radiol. Endodontol..

[12] Epstein J.B., Stevenson-Moore P. (2001). Periodontal disease and periodontal management in patients with cancer. Oral Oncol..

[13] Feyizoglu B.B., Aysesek B.H., Kaya S., Baser U., Olgac V., Isik A.G. (2025). Effects of orthodontic tooth movement on periodontal tissues after ligature-induced periodontitis through the mechanism of RANKL-induced osteoclastogenesis: An animal study. BMC Oral Health.

[14] Kilkenny C., Browne W.J., Cuthill I.C., Emerson M., Altman D.G. (2010). Improving bioscience research reporting: The ARRIVE guidelines for reporting animal research. PLoS Biol..

[15] de Molon R.S., Park C.H., Jin Q., Sugai J., Cirelli J.A. (2018). Characterization of ligature-induced experimental periodontitis. Microsc. Res. Tech..

[16] Sønstevold T., Johannessen A.C., Stuhr L. (2015). A rat model of radiation injury in the mandibular area. Radiat. Oncol..

[17] Marx R.E. (1983). Osteoradionecrosis: A new concept of its pathophysiology. J. Oral Maxillofac. Surg..

[18] Irie M.-S., Mendes E.M., Borges J.S., Osuna L.G.G., Soares P.B.F. (2018). Periodontal therapy for patients before and after radiotherapy: A review of the literature and topics of interest for clinicians. Med. Oral Patol. Oral Cir. Bucal.

[19] Güll F.D., Deppe H., Kesting M., Schwarzer C. (2017). Periodontal disease-like bone loss after adjuvant radiotherapy in the head and neck region: A case report and review of the literature. Quintessence Int..

[20] Köse O., Arabaci T., Kizildag A., Erdemci B., Özkal Eminoğlu D., Gedikli S., Özkanlar S., Zihni M., Albayrak M., Kara A. (2017). Melatonin prevents radiation-induced oxidative stress and periodontal tissue breakdown in irradiated rats with experimental periodontitis. J. Periodontal Res..

[21] Giannopoulou C., Martinelli-Klay C.P., Lombardi T. (2012). Immunohistochemical expression of RANKL, RANK and OPG in gingival tissue of patients with periodontitis. Acta Odontol. Scand..

[22] Cochran D.L. (2008). Inflammation and bone loss in periodontal disease. J. Periodontol..

[23] Chan K.C., Perschbacher S.E., Lam E.W.N., Hope A.J., McNiven A., Atenafu E.G., Lee L., Pharoah M.J. (2016). Mandibular changes on panoramic imaging after head and neck radiotherapy. Oral Surg. Oral Med. Oral Pathol. Oral Radiol..

[24] Alwood J.S., Shahnazari M., Chicana B., Schreurs A.S., Kumar A., Bartolini A., Shirazi-Fard Y., Globus R.K. (2015). Ionizing radiation stimulates expression of pro-osteoclastogenic genes in marrow and skeletal tissue. J. Interferon Cytokine Res..

[25] Wu X., Pan G., McKenna M.A., Zayzafoon M., Xiong W.C., McDonald J.M. (2005). RANKL regulates Fas expression and osteoclast apoptosis. J. Bone Miner. Res..

[26] de Almeida J.M., Ervolino E., Bonfietti L.H.F.S., Novaes V.C.N., Theodoro L.H., Fernandes L.A., Martins T.M., Faleiros P.L., Garcia V.G. (2015). Adjuvant therapy with sodium alendronate for the treatment of experimental periodontitis in rats. J. Periodontol..

[27] Morita M., Uehara O., Kawabata Y., Imai K., Fujita T., Shiba H., Kurihara H. (2016). Elevation of pro-inflammatory cytokine levels following anti-resorptive drug treatment is required for osteonecrosis development in infectious osteomyelitis. J. Bone Miner. Res..

[28] Kim Y.D., Kim S.S., Hwang D.S., Kim S.G., Kim W.K., Kim J.R., Shin S.H. (2009). Expression of receptor activator of nuclear factor-κB ligand, receptor activator of nuclear factor-κB, and osteoprotegerin following low-level laser treatment on deproteinized bovine bone graft in rats. J. Periodontal Implant Sci..

[29] Sroussi H.Y., Epstein J.B., Bensadoun R.-J., Saunders D.P., Lalla R.V., Migliorati C.A., Heaivilin N., Zumsteg Z.S., Zhang L., Brennan M.T. (2017). Common oral complications of head and neck cancer radiation therapy. Cancer Med..

